# The management of acetabular malunion with traumatic arthritis by total hip arthroplasty 

**DOI:** 10.12669/pjms.291.2900

**Published:** 2013

**Authors:** Qing-jiang Pang, Xiao Yu, Xian-jun Chen, Zhen-chun Yin, Guo-zhong He

**Affiliations:** 1 Qing-jiang Pang, Department of Orthopaedics Centre, Ningbo No.2 Hospital, Ningbo, 315010. China.; 2 Xiao Yu, Department of Orthopaedics Centre, Ningbo No.2 Hospital, Ningbo, 315010. China.; 3 Xian-jun Chen, Department of Orthopaedics Centre, Ningbo No.2 Hospital, Ningbo, 315010. China.; 4 Zhen-chun Yin, Department of Orthopaedics Centre, Ningbo No.2 Hospital, Ningbo, 315010. China.; 5 Guo-zhong He, Department of Orthopaedics Centre, Ningbo No.2 Hospital, Ningbo, 315010. China.

**Keywords:** Acetabular fracture, Traumatic arthritis, Total hip arthroplasty, Harris scoring system

## Abstract

***Objective:*** To explore the treatment strategies and clinical effect of the acetabular malunion with traumatic arthritis by total hip arthroplasty.

***Methodology:*** A retrospective analysis was conducted on 47 cases of acetabular malunion with traumatic arthritis from June 2000 to December 2009. All the patients underwent total hip arthroplasty with bone grafting or titanium cage for bone defect of the acetabulum. Harris hip scoring system was used for evaluating the functional recovery of the hip joint.

***Results:*** Thirty three cases had an average of 47 months follow-up. No prosthesis was loosened and the function of hip joint was improved obviously with the Harris hip scores improving from 43.5 to 87.6. However, there were one case of sciatic nerve injury and four cases of heterotopic ossification postoperatively.

***Conclusion:*** Total hip arthroplasty might be an effective treatment of acetabular malunion with traumatic arthritis. Proper evaluation and reasonable reconstruction of acetabular defect as well as reasonable selection of prosthesis are essential to obtain an excellent outcome.

## Introduction

 The uncertainty of operative results for severely displaced acetabular fractures and the complications of nonoperative management are the key reasons to the acetabular malunion with traumatic arthritis.^1^ Once the severe acetabular malunion with traumatic arthritis causes the necrosis of the femoral head and the malalignment of the lower limb; it will result in abnormal gait and dysfunction of the hip joint.^2^ Thus, the acetabular malunion may be life-altering to the patients for not only a great symptomatic effect but also a huge economic burden. Therefore, once a failed open reduction of internal fixation (ORIF) of acetabular fracture occurs, a revision surgery is always needed to prevent the vicious cycle of pathological changes as far as possible. Re-ORIF to the failed acetabular fracture was once deemed as a considerable way for the revision surgery. However, both the surgeons and patients should know that although the revision surgery may result in a more functional hip joint, there is still considerable disability as indicated by lower functional scores on the 36-Item Short Form Health Survey (SF-36) and Harris Survey, especially to the patient of acetabular malunion with traumatic arthritis. ^3^

Currently, with the development of the total hip arthroplasty (THA), it had been attempted to the acetabular malunion with traumatic arthritis. Various successful cases indicated that it could be an alternative measure to improve the function of the hip joint.^4^^,^^5^ However, it often encounters a challenge on how to reconstruct a stable and functional hip joint during the operations.^6^ We believe that bone grafting or titanium cage might be a feasible way for acetabulum reconstruction.

 Our objective was to explore the treatment strategies and clinical effect of the acetabular fracture malunion with traumatic arthritis by total hip arthroplasty with bone grafting or titanium cage for the bone defect of the acetabulum.

## Methodology

***Patients and methods: ***The study was conducted on 47 cases of acetabular malunion with traumatic arthritis in our center (37 males and 10 females at the age of 48.5 years, ranged from 31 to 62 years), whose initial management were failed either by conservative treatment or ORIF.

 All the 47 cases were injured in high-energy trauma and according to the Letounel classification, there were 10 cases of transverse acetabular fracture, 11 cases of posterior column and posterior wall fractures, 12 cases of transverse and posterior wall fractures with central dislocation of the femoral head, 8 cases of both-column fractures and 6 cases of T-shaped fracture. Initial management including conservative treatment (12 cases) and ORIF (35 cases) were performed in these patients; however, bad prognosis of acetabular malunion with traumatic arthritis occurred in all the patients, among which 6 cases were secondary to femoral head necrosis simultaneously. Furthermore, all the cases had a certain degree of limb shortening from 1.0cm to 4.5cm (2.65 cm on average). All the cases underwent THA. The mean time from acetabular fracture to THA was 67.5 months (ranged from 15 to 216 months).

 Preoperative preparation for these patients should be individualized since different patterns of acetabular fractures and defect may need different ways for reconstruction. Therefore, X-ray of pelvic by anteroposterior view, obturator oblique view and iliac oblique view together with three-dimensional reconstruction of CT must be prepared to evaluate the acetabular defect and determine the reasonable reconstruction way. Erythrocyte sedimentation rate (ESR) and C-reactive protein (CRP) should also be examined conventionally. Only when the ESR< 30mm/h and the CPR< 5mg/L, can the THA be performed.

***Operative techniques: ***The THA began with the posterolateral approach in all cases with lateral position. Attention should be paid to protect the gluteus medius muscle. Meanwhile, the scar tissue or granulation tissue that impacted the exposure of the acetabulum should be eliminated thoroughly. The assessment of the acetabular defect was based on the AAOS classification system, which includes 11 cases of cavity-pattern, nine cases of segmental-pattern and 27 cases of mixed-pattern. Of all the 35 patients treated with ORIF initially, six cases had the difficulty in placing the acetabular prosthesis due to the position of the screws; therefore, local drilling was performed to remove the screws and five cases benefited from this technique. While the other one, whose screws were difficult to remove, were treated with drilling shorter of the screws and covering the granular autogenous bone. Furthermore, there were another nine patients, whose plates were partly exposed in the acetabulum and the coverage of the plates by bone grafting with granular autogenous femoral head or allogeneic bones were performed successfully. For other patients, their implants were preserved since they did not affect the placement of acetabular prostheses.

**Fig.1:**
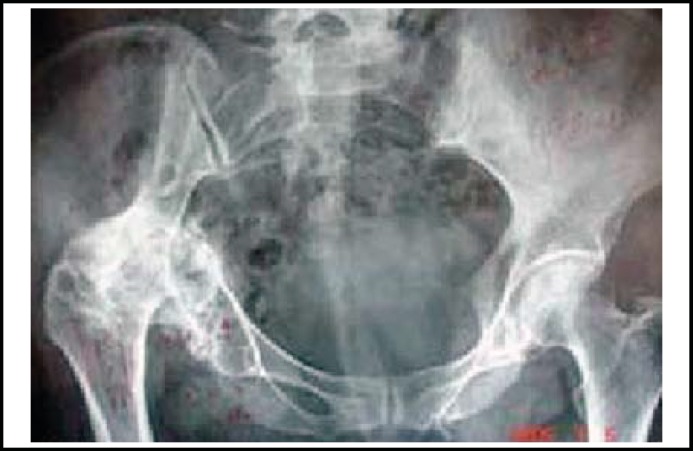
A 65 years old female suffered from acetabular malunion with traumatic arthritis after conservative treatment for 8 years.

**Fig.3:**
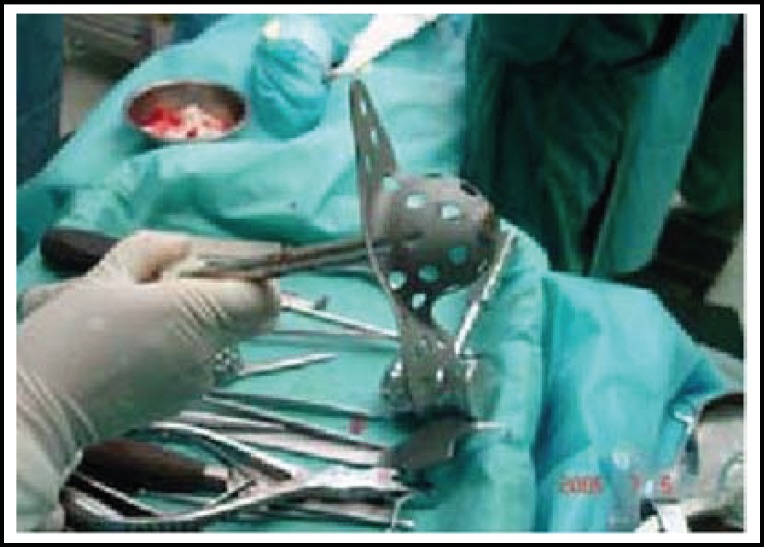
The Burch-Schneider acetabular cage used in surgery.

**Fig.4:**
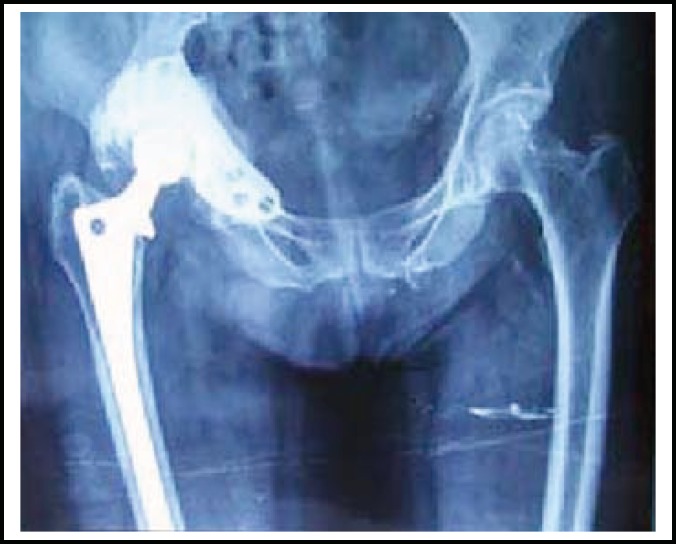
X-ray of pelvic by anteroposterior view showed the reconstructed acetabulum with cage after 3 years of the total hip arthroplasty.

The acetabular defect could be treated with bone grafting or titanium cage.^7^ We treated eight cases with structural and granular bone grafting of autogenous femoral head, 16 cases with simplex granular bone grafting of autogenous femoral head, six cases with granular bone grafting of autogenous femoral head and allogeneic bones and five cases with titanium acetabular cage to reconstruct the acetabulum (Burch-Schneider cage™, Zimmer Inc, Warsaw, IN, USA). The diameters of the granular bone ranged from 5mm to 10mm and the structural grafted bones were fixated by screws. (Fig. 1-4). Of all the cases, the acetabular prostheses were fixed by bone cement in 13 cases and the other 34 cases were fixated without cement. The femoral prostheses were fixed without cement in all cases.

***Postoperative management: ***All the patients accepted physical therapy such as foot pump as soon as the operations were over. Low molecular weight heparin was emphasized to be used for at least 10 days to prevent deep venous thrombosis (DVT) after removal of the drainage tube. Patients were asked to start non-weight-bearing activities with the help of the walker 3-5 days postoperatively and partial weight-bearing activities 4-6 weeks later. About 10~12 weeks later, the patients could walk by crutches. However, full-weight-bearing activities were prohibited until the grafting bone for the acetabular defect healed completely.

***Clinical and radiological analysis: ***The patients were asked to be followed up at the first year for three times (the 3rd month, the 6th month and the 12th month postoperatively) and after that the times of follow-up could be reduced to once a year. The content of follow-up includes the Harris scores of the hip joints, the length of the limbs and the X-ray of the pelvis films. The criterion of Russoui-Harris is used to evaluate the displacements of the acetabular prostheses and the variation of the acetabular angle.^8^ The loosening of acetabular prosthesis is defined as over 4mm of the displacements in any directions and over 4º of the variation of acetabular abduction angle. A Student’s *t*-test was used to compare the differences of Harris scores before and after surgery. A *P*-value<0.05 was considered to be statistically significantly.

**Table-I:** Statistical description of acetabular malunion with traumatic arthritis classification, surgical management and outcomes.

*Variable*	*Number/Mean± SD*
Age (years)	48.5**±**16.2
Gender(male/female)	37/10
Acetabular fracture (Letounel classification)	
Transverse acetabular fracture	10
Posterior column and posterior wall fractures	11
Transverse and posterior wall fractures	6
Both-column fractures	8
T-shaped fracture	6
Average preoperative limb shortening(cm)	2.65
Shortening<2cm	19
Shortening>2cm	28
Acetabular defect (AAOS classification)	
Cavity-pattern	9
Segmental-pattern	11
Mixed-pattern	27
Methods of acetabular reconstruction	
Structural and granular autogenous bone grafting	8
Simplex granular autogenous bone grafting	16
Granular autogenous and allogeneic bone grafting	6
Titanium acetabular cage	5
Management of previous implants	
Local drilling to remove the screws	5
Drilling shorter and covered by bone grafting	1
Covered by bone grafting (plates)	9
Preserved	20
Selection of the prosthesis	
Cement acetabular prosthesis	13
Cementless acetabular prosthesis	34
Cement femoral prosthesis	47
Average postoperative Harris scores (33 cases)	87.6±7.2
Excellent (90-100)	7
Good (80-89)	16
Fair (70-79)	8
Poor (<70)	2
Average postoperative limb lengthening(cm)	2.3
Position of the acetabular rotational center	
Restored	27
Moved inward	6
Complications	
Sciatic nerve injury	1
DVT	8
Heterotopic ossification	4

## Results

 The mean operative time of these 47 patients was 156 minutes and the mean blood loss was 750ml. A total of 33 cases had an average of 47 months follow-up (ranged from 15 to 72 months). Of all the 33 cases, sciatic nerve injury occurred in one case and recovered completely by neurotrophic treatment for four months. DVT with lower limbs swelling and pain occurred in eight cases and healed gradually with Low molecular weight heparin for 7 to 28 days. The heterotopic ossification of Brooker I occurred in three cases and Brooker III in one case after three months of the surgery, however, no further progress was found in the later period of the follow-up. Postoperatively, the function of the hip joint also improved obviously with the Harris scores rising from preoperative 43.5±6.7 to 87.6±7.2 (evaluated at 12th month postoperatively). Student’s *t*-test by SPSS 13.0 (SPSS Inc., Chicago, IL, USA) showed the significantly statistical differences in Harris Scores (*t*=3.10, *p*<0.05). Preoperatively, there was an average of 2.65cm shortening of the limb, while the length increased at an average of 2.3cm postoperatively. The rotational center of the acetabulum was restored in 27 cases, however, the center moved inward in six cases on X-ray. The acetabular prostheses and femoral prostheses were fixated firmly without signs of loosening and osteolysis. (Table-I)

**Figure:**
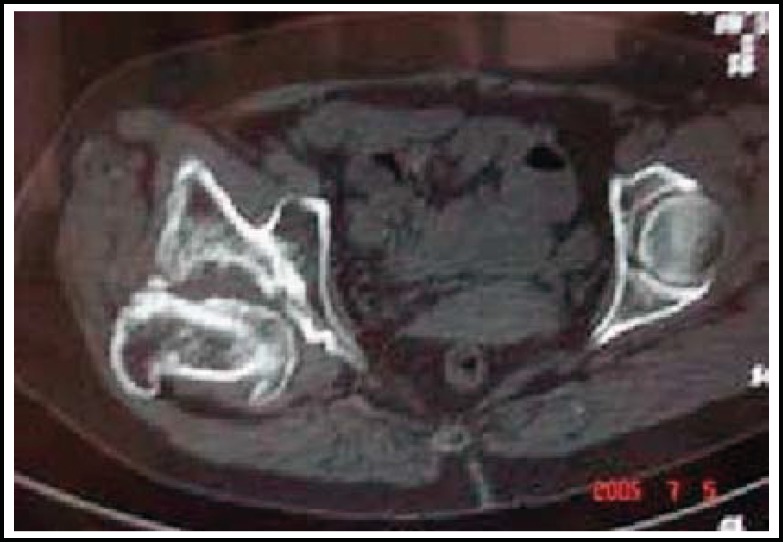
Fig.2 CT scan showed the acetabular defect in posterior column and posterior wall.

## Discussion

 In general, the clinical outcomes of THA for acetabular fractures are often not as good as the osteoarthritis because of the higher rate of the unexpected complications.^9^ Furthermore, another important reason for THA not being in favor by most scholars is that it is very difficult to guarantee the stability of the acetabular prosthesis in a fractured acetabulum. Therefore, it almost becomes the consensus that THA for acetabular fracture is only indicated in dysfunction of the hip joint due to the traumatic arthritis or femoral head necrosis. However, THA for early stage of acetabular fracture is also favored by some scholars, however, strict indications should be followed^10^^,^^11^, which includes the following five conditions: □ Patients over 65 years of age with severe osteoporosis; □ Serious osteoarthritis of the hip joint before acetabular fracture; □ Concomitant femoral neck fracture or splitting femoral head fracture; □ Intra-articular comminuted fracture with more than 10 bone blocks; □Compacted femoral head or over 40% of the subsidence of the acetabular surface; Occasionally, THA for late stage of acetabular fracture is also performed with the indications we summarized as follows: □ Revision for failed ORIF of acetabular fracture; □ Traumatic osteoarthritis after acetabular fracture; □ Femoral head necrosis and collapse; □ Subluxation or dislocation of the hip joint.

 THA for acetabular malunion with traumatic arthritis is time- consuming and complicated. Therefore, detailed preoperative preparation should be performed including the ESR, CRP to rule out the inflammation state. X-ray of pelvic by anteroposterior view, obturator oblique view and iliac oblique view must be examined to comprehend the contour of the acetabulum, while three-dimensional reconstruction of CT is essential to evaluate the acetabular defect and the position of the previous implants.^12^ With the help of these examinations, the detailed preoperative plans such as the surgical approach, the management of the previous implants, the reconstruction way of acetabulum defect, the selection of the prosthesis and the prevention of the postoperative complications could be made.

 Previous implants commonly exist in these patients. When they are treated with THA, the removal of the implants may cause the potential risk of the iatrogenic injuries of both the bony structure of acetabulum and the neurovascular bundles.^13^ Therefore, it is not necessary to remove the previous implants if they do not interfere with the placement of the acetabular prosthesis. If the implants are exposed in the acetabulum and difficult to be removed, the bone grafting to cover the implants and / or the trimming of the implants may be applicable. In this study, we encountered the difficulty in placing the acetabular prosthesis in six cases, which were managed by local drilling to remove the screws in five cases and covering the particles of autogenous bone in one case. Furthermore, we encountered another nine cases with the plates being partly exposed in the acetabulum and we chose to cover the plates by bone grafting with granular autogenous femoral head or allogeneic bones. For other patients, we chose to preserve the implants without any interference since they did not affect the placement of acetabular prostheses.

 Acetabular defect is always a thorny problem to reconstruct the acetabulum and place the acetabular prosthesis. Bone grafting may be an advisable method to deal with it.^14^^,^^15^ For the patients of small cavity-pattern acetabular defect (<25mm), granular autogenous bone grafting (5~10mm cancellous particles) from femoral head could be used. While for the cases of large cavity-pattern defect (>25mm), it is advised to use the autogenous femoral head or allogeneic bone to perform the structural bone grafting.^16^ As for the management of segmental-pattern acetabular defect, we summarize that if the defect involves only a part of the acetabular rim that almost does not affect the stability of prosthesis, it is not necessary to be treated with bone grafting. However, if defect involves the acetabular roof and the anterior and posterior wall, structural bone grafting might be needed. Occasionally, the titanium cage, which are often used in the revision surgery of THA, could be used to reconstruct the acetabulum if the defect largely involved the acetabular roof, posterior wall and posterior column.^17^ In this study, eight patients were treated with structural and granular bone grafting of autogenous femoral head, 16 cases with simplex granular bone grafting of autogenous femoral head, six cases with granular bone grafting of autogenous femoral head and allogeneic bones and five cases with titanium acetabular cage to reconstruct the acetabulum. In order to obtain a firm bone bed for the placement of the acetabular cage, the structural bones should be fixated by screws and the granular bones should be packed by SLooff-Ling techniques.^18^

 The reasonable selection of prosthesis is another important factor to the success of the surgery. The surgeons is always in a dilemma on how to select an optimal prosthesis to an individual. In fact, the selection of prosthesis must be based on the comprehensive evaluation of the bone quality, the age, the ways of the bone grafting and fixation.^19^ In general, cement-prosthesis is frequently considered to be used for the patients over 65 years old, while cementless-prosthesis can be used in patients with over 50% of the effective contact area between the acetabular prosthesis and the acetabulum. However, if the effective contact area < 50%, the fixation of the cement-prosthesis in the acetabulum, which is reconstructed by structural bone grafting and titanium cage, might be the optimal method.^20^ As for the cases of type III and type IV acetabular defect, which is reconstructed by the structural allogeneic bone grafting and augmented titanium cage, it is best to select the cement-prosthesis.^21^ In this study, acetabular cement-prosthesis was used in 13 cases and cementless-prosthesis was used in 34 cases. The femoral cement-prosthesis was used for all the cases.

## Conclusions

 Total hip arthroplasty might be an effective treatment for acetabular malunion with traumatic arthritis; however, it should be treated seriously due to the difficult exposure and high rate of the complications. One of the most difficult procedures in the operation is how to replenish the acetabular defect to reconstruct a stable and normal anatomic artificial acetabulum. Since different individuals may exhibit the different patterns of the acetabular defects, it is emphasized that individualized preoperative assessment and treatment programs should be performed. Proper evaluation and reasonable reconstruction of acetabular defect as well as reasonable selection of prosthesis are all essential to obtain an excellent outcome.
